# Presence of herpesviruses, parvoviruses, and polyomaviruses in sinonasal lymphoma

**DOI:** 10.1007/s00405-024-08702-0

**Published:** 2024-05-17

**Authors:** Maria K. Jauhiainen, Ushanandini Mohanraj, Maria F. Perdomo, Jaana Hagström, Caj Haglund, Antti A. Mäkitie, Maria Söderlund-Venermo, Saku T. Sinkkonen

**Affiliations:** 1 Department of Otorhinolaryngology, Head and Neck Surgery, Head and Neck Center, Helsinki University Hospital and University of Helsinki, POB 263, 00029 HUS Helsinki, Finland; 2 Department of Virology, University of Helsinki and Helsinki University Hospital, Helsinki, Finland; 3 Research Program in Systems Oncology, Faculty of Medicine, University of Helsinki, Helsinki, Finland; 4 The Doctoral Programme in Clinical Research, Faculty of Medicine, University of Helsinki, Helsinki, Finland; 5 Department of Pathology, University Hospital of Helsinki, Helsinki, Finland; 6 Department of Oral Pathology and Radiology, University of Turku, Turku, Finland; 7 Division of Ear, Nose and Throat Diseases, Department of Clinical Sciences, Intervention and Technology, Karolinska Institute and Karolinska University Hospital, Stockholm, Sweden; 8 Translational Cancer Research Medicine, Research Programs Unit, Faculty of Medicine, University of Helsinki, Helsinki, Finland; 9 Department of Surgery, Helsinki University and Helsinki University Hospital, Helsinki, Finland

**Keywords:** DNA viruses, Lymphoma, Paranasal sinuses, Herpesvirus 4, Human, Parvovirus

## Abstract

**Purpose:**

Sinonasal lymphoma (SL) is a rare lymphatic neoplasm of the nasal cavities, paranasal sinuses and nasopharynx. Whereas some risk factors for SL subtypes have been identified, their aetiology is unknown. Along with other predisposing factors, the viral association of lymphomas, such as Epstein-Barr virus (EBV) and Burkitt and Hodgkin lymphomas, is well-established. Modern molecular biology techniques have enabled the discovery of novel human viruses, exemplified by the protoparvovirus cutavirus (CuV), associated with cutaneous T-cell lymphoma. These findings, and the anatomical location of the sinonasal tract with its rich microbiome and infectious agents, justify in-depth studies among SL.

**Methods:**

We analysed the presence of 20 viruses of *Orthoherpesviridae*, *Parvoviridae*, and *Polyomaviridae* by qPCR in 24 SL tumours. We performed RNAscope in situ hybridisation (RISH) to localize the viruses. Parvovirus-specific IgG was analysed by enzyme immunoassay and targeted next-generation sequencing (NGS) was applied to detect CuV in plasma.

**Results:**

We detected viral DNA in 15/24 (63%) tumours; nine of EBV, six of human herpesvirus (HHV) -7, four each of HHV-6B and parvovirus B19, two of cytomegalovirus, and one each of CuV and Merkel-cell polyomavirus. We found tumours with up to four viruses per tumour, and localized CuV and EBV DNAs by RISH. Two of the ten plasma samples exhibited CuV IgG, and one plasma sample demonstrated CuV viremia by NGS.

**Conclusion:**

Viruses were frequent findings in SL. The EBV detection rate was high in diffuse large B-cell lymphoma, and co-detections with other viruses were prevalent.

## Introduction

Sinonasal lymphomas (SLs) form a heterogenous entity of lymphomas of a specific anatomical location, the sinonasal tract. The classification of lymphomas follows the 5th edition of the WHO Classification of Haematolymphoid Tumours (WHO- HAEM5). Diffuse large B-cell lymphoma (DLBCL) is the most common subtype followed by extranodal natural killer/T-cell lymphoma (ENK/TL) with geographical variation in prevalence [1, 2].

Microbes cause approximately 15% of all cancers worldwide [3]. The anatomical location of the sinonasal tract is exposed to an array of microbes. Within malignant tumours of the upper respiratory tract, viruses have an evident etiological role, such as the associations of human papillomavirus (HPV) with oropharyngeal squamous cell carcinoma and Epstein-Barr virus (EBV) with lymphatic tumours, such as Burkitt lymphoma, Hodgkin lymphoma, and DLBCL [4–6]. Additional risk factors for lymphomas include human immunodeficiency virus (HIV), human herpesvirus-8 (HHV-8), hepatitis B and C viruses, and human T-lymphotropic virus, as well as a family history of lymphomas, immunological disorders, ionizing radiation, agricultural pesticides, and increased body-mass index in young adults [2, 7].

In addition to viruses of *Papillomaviridae* and *Orthoherpesviridae*, oncogenic viruses are found within *Polyomaviridae* (Merkel cell polyomavirus, MCPyV, the cause of Merkel cell carcinoma), and *Hepadnaviridae* (hepatitis B virus in liver cancer) [8, 9]. Oncolytic viruses, in turn, are found within *Parvoviridae* [10].

Many new parvoviruses have been detected in the recent decade, and their behaviour and role are under stringent investigation [11]. Cutavirus (CuV), discovered in 2016, has been associated with cutaneous T-cell lymphoma and its precursor parapsoriasis, which makes it interesting in the context of SL [12–17]. In addition to CuV, the *Protoparvovirus* genus includes human bufavirus (BuV) and tusavirus (TuV), and the *Bocaparvovirus* genus includes the human bocaviruses 1–4 (HBoV1-4) [11, 18, 19].

Other herpesviruses, such as HHV-6 and -7 are widely distributed especially in the gastrointestinal tract and its organs, as well as in the salivary glands [20, 21]. The latent viruses harboured by monocytes, macrophages and CD4+ T lymphocytes, have a unique feature of being able to modify the host-cell immune response, possibly facilitating the effects of other agents or viruses on these cells [20–22].

Whereas many herpes-, parvo-, and polyomaviruses are ubiquitous within the general population, the persistence and presence in various tissues and tumours vary [11, 23, 24]. Given the interesting anatomical locus with its rich microbiome, we present a study focusing on SL with the aim to screen for a broad range of DNA viruses not studied before in this context, including “new” viruses detected in the recent decade.

## Materials and methods

### Ethics

The research ethics committee at the Helsinki University Hospital approved the study (§31/07.03.2019) and a research permission was granted (HUS/332/2019).

### Patients and clinical specimens

This was a retrospective cross-sectional study. The patients were treated at the Departments of Otorhinolaryngology—Head and Neck Surgery and Oncology, Helsinki University Hospital, Helsinki, Finland. We recorded the clinical information from hospital charts (Table 1).

**Table 1:** Patient samples and clinical information

Patient code	Diagnosis (y)	Age (y) / sex F/M	Lymphoma type**	Tobacco Yes/No/ No more	Alcohol missuse Yes/No	Ann-Arbor (I-IV)***	MIB-1 (%)****	Location: Naso- pharynx	Location: Paranasal sinuses	Location: Nasal cavity	Comorbidity
**L11**	**2015**	**65/M**	**MCL**	**No more**	**No**	**IA**		**1**			**Alcohol**
L12	1987	78/F	Small lymphocytic (B-cell)	N/A	N/A	N/A	N/A	N/A	N/A	N/A	N/A
L13	1988	86/F	Centroblastic/ -cytic or T-cell	N/A	N/A	N/A	N/A	N/A	N/A	N/A	N/A
L14/15*	**1992**	**58/F**	**DLBCL-NOS**	**N/A**	**N/A**	**N/A**	**N/A**	**N/A**	**N/A**	**N/A**	**N/A**
L16	1992	74/F	DLBCL-NOS	N/A	N/A	N/A	N/A	N/A	N/A	N/A	N/A
**L17**	**1993**	**82/M**	**Centroblastic /** **diffuse large B cell**	**N/A**	**N/A**	**N/A**	**N/A**	**N/A**	**N/A**	**N/A**	N/A
**L18**	**1995**	**63/M**	**DLBCL-NOS**	**N/A**	**N/A**	**N/A**	**N/A**	**N/A**	**N/A**	**N/A**	**T–cell lymphoma**
L19	1996	72/M	DLBCL-NOS	N/A	N/A	N/A	N/A			1	N/A
L20	1999	81/F	DLBCL-NOS	N/A	N/A	N/A	N/A	N/A	N/A	N/A	Ca epidermoides (oral)
L21/22*	2000	46/M	DLBCL-NOS	N/A	N/A	N/A	N/A	1	1		HIV + , Rectum adenocarcinoma
L23	2001	73/M	DLBCL-NOS	Yes	N/A	N/A			1		
L24	2002	86/F	B-cell	N/A	N/A	N/A	N/A	N/A	N/A	N/A	N/A
**L25**	**2003**	**39/M**	**Anaplastic large** **cell (ALK?)**	N/A	N/A	**IIAE**	**MIB 60**	**1**			**–**
L26	2004	60/F	DLBCL-NOS	N/A	N/A	IVB	MIB 80		1		–
**L27**	**2004**	**46/M**	**DLBCL-NOS**	**N/A**	**No**	**IIIA**	**MIB > 90**	**1**			**HIV + , HCV + **
**L28**	**2007**	**34/M**	**DLBCL-NOS**	**N/A**	**No**			**1**			**AIDS**
**L29**	**2011**	**58/F**	**DLBCL-NOS**	**No more**	**N/A**	**IIAE**	**MIB 50**		**1**		**–**
**L30**	**2012**	**58/M**	**DLBCL-NOS**	**Yes**	**No**	**IIAE**		**1**	**1**	**1**	**–**
**L31**	**2014**	**70/M**	**DLBCL-NOS, BCL-2 rearrangement**	**No more**	**N/A**	**IA**	**MIB 50**	**1**			**–**
**L32**	**2016**	**67/F**	**MCL**	**Yes**	**No**	**IVA**	**MIB 70**	**1**			**–**
**L33**	**2017**	**64/F**	**DLBCL-NOS**	**Yes**	**No**	**IVA**		**1**			**–**
**L34**	**2018**	**64/F**	**DLBCL-NOS**	**No more**	N/A	**IIAE**	**MIB 90**	**1**			**–**
**L35**	**2018**	**72/M**	**DLBCL-NOS**	**Yes**	**No**	**N/A**	**N/A**	**1**			**Lung carcinoma**
**L36**	**2017**	**84/F**	**DLBCL-NOS**	**N/A**	**N/A**	**IIIA**	**MIB 90**	**1**			**–**

*MCL* Mantle cell lymphoma, *DLBCL-NOS* diffuse large B-cell lymphoma – not otherwise specified, *N/A* not available

The location of the lymphoma is sinonasal, but no further details were available

Bolded samples were virus-DNA positive

^*^The same results were obtained from two samples of the same patient

^**^Classified according to WHO- HAEM5, when possible

^***^Ann-Arbor classification

^****^MIB-1 cell proliferation marker N/A, not available

We collected FFPE tumours representing 24 SL patients diagnosed during 1987–2018 from the Helsinki Biobank (permission no §73/15.05.2019, HUS/118/2019). The Helsinki Biobank provided plasma samples from seven SL patients with available FFPE tumour samples and additionally from three SL patients with no tumour samples available.

We analysed all tumours by qPCR for viral DNA, and the high-load ones by RNAscope in situ hybridisation (RISH) for cell tropism. We collected plasma samples to investigate the serological status of parvoviruses B19V, BuV, CuV and TuV, and one underwent targeted NGS.

### DNA extraction

All tumour biopsies were collected in a PCR-sterile manner from FFPE tissue blocks as 2-mm punch biopsies in sterile 1.5 ml microcentrifuge tubes. The correct localization was evaluated microscopically from the representative tissue slide by the first author, and whenever in doubt, confirmed by the senior pathologist. If enough material were available, two distinct biopsies from the tumour were taken. Prior to puncturing, new HE slices were cut from paraffin blocks to ensure the punching to hit tumour tissue. DNA was extracted with QIAamp DNA FFPE Tissue Kit (Qiagen, Heiden, Germany), according to the manufacturer’s protocol, with slight modifications: the paraffin treatment with xylene was done twice and 40 µl of proteinase K was used. We eluted the DNA preps in 60 µl AE buffer and stored them at -20°C. From one plasma sample (patient L31), we extracted DNA with QIAamp DNA blood Mini Kit (Qiagen), according to the manufacturer’s protocol. The DNA yields and human cell quantity were evaluated by comparing viral loads with that of the human reference gene *RNase P* by qPCR [25].

### Virus DNA detection by qPCR

The viruses analysed with qPCR included the herpesviruses (herpes simplex virus-1 and -2, varicella zoster virus, EBV, cytomegalovirus (CMV), HHV-6A, HHV-6B, HHV-7, and HHV-8), the polyomaviruses (BK polyomavirus (BKPyV), JC polyomavirus (JCPyV), and MCPyV), and the parvoviruses (B19V, BuV, CuV, TuV, and HBoV1–4). Details are listed in Table 2 [14, 25–28]. All qPCR reactions contained either Maxima probe qPCR Master Mix (Thermo Fischer Scientific, Pittsburgh, PA, USA) or TaqPath ProAmp master mix Thermo Fisher), as described [29]. We performed all real-time qPCR assays with AriaMx Realtime PCR System (Agilent Technologies, Santa Clara, CA, USA).

**Table 2:** Viruses analysed by qPCR and NGS, and qPCR primer and probe sequences

Detection method	qPCR	NGS
Family		
*Orthoherpesviridae*	Herpes simplex 1	Herpes simplex 1
	Herpes simplex 2	Herpes simplex 2
	Varicella zoster	Varicella soster
	Epstein Barr virus	Epstein-Barr virus
	Cytomegalovirus	Cytomegalovirus
	Human herpesvirus 6A	Human herpesvirus 6
	Human herpesvirus 6B	
	Human herpesvirus 7	Human herpesvirus 7
	Kaposi’s Sarcoma virus	Kaposi’s Sarcoma virus
*Polyomaviridae*	BK polyomavirus	BK polyomavirus
	JC polyomavirus	JC polyomavirus
	Merkel cell polyomavirus	KI polyomavirus
		WU polyomavirus
		Merkel cell polyomavirus
		Human polyomavirus 6
		Human polyomavirus 7
		TS polyomavirus
		Human polyomavirus 9
		MW polyomavirus
		STL polyomavirus
		Human polyomavirus 12
		NJ polyomavirus
		Simian virus 40
*Parvoviridae*	Human parvovirus B19	Human parvovirus B19
	Human bocavirus 1	Human bocavirus 1
	Human bocavirus 2	Human bocavirus 2
	Human bocavirus 3	Human bocavirus 3
	Human bocavirus 4	Human bocavirus 4
	Cutavirus	Cutavirus
	Tusavirus	
	Bufavirus	
*Papillomaviridae*		Human papillomavirus type 2
		Human papillomavirus type 6
		Human papillomavirus type 11
		Human papillomavirus type 16
		Human papillomavirus type 18
		Human papillomavirus type 31
		Human papillomavirus type 45
*Hepadnaviridae*		Hepatitis B virus
*Anelloviridae*		Torque teno virus (1, 10, 13)
*Poxviridae*		Variola minor & major virus

QPCR, quantitative polymerase chain reaction; NGS, next-generation sequencing; EBV, Epstein Barr virus; HHV-7, Human herpesvirus 7; B19 and B19V, Human parvovirus B19; HHV-6B, Human herpesvirus 6B; CMV, cytomegalovirus; CuV, cutavirus; MCPyV, Merkel cell polyomavirus; EIA, enzyme immunoassay; BuV, bufavirus; TuV, tusavirus

We included molecular biology grade water in all qPCR reactions as a non-template control and used ten-fold diluted plasmids (10^1^–10^6^), containing each viral target regions as qPCR standards and as positive controls.

### Virus DNA detection by NGS

We processed one plasma sample of patient L31, in whose tissue sample we had detected high copy numbers of CuV DNA, by targeted NGS, given the unknown nature of this rather newly discovered virus. The viruses targeted with the NGS are listed in Table 2. We fragmented the DNA mechanically and prepared the libraries with unique double indexes and performed in solution capture-targeted enrichment of the viral DNAs [29, 30]. The enriched library was sequenced, at the FIMM genomics unit of the University of Helsinki, with Novaseq 6000 (S1, PE151 kit; Illumina). The pipeline was validated as described in Pratas et al. [31] and has been tested on a wide range of materials, including human tissues, bone, serum/plasma, and formalin-fixed samples [24]. A blank extraction control was carried downstream alongside the sample throughout the entire process, spanning library preparation, enrichment, and sequencing.

### NGS data analysis

The data were analysed with TRACESPipeLite, a fully automatic program [30–32]. When in low breadth coverage (< 15%), we manually inspected and confirmed the individual reads by BLAST. The viral reads of cutavirus were manually verified using BLAST and confirmed to match exclusively cutavirus.

### In situ hybridisation

We chose five tumours for RISH; (Advanced Cell Diagnostics (ACD), Newark, CA), each representing the highest virus DNA load (3.45 × 10^2^–2.08 × 10^6^ cpm) of either CuV, EBV, HHV-6B, HHV-7 or B19V, and five additional virus DNA-negative SL tumours. We performed RISH for the CuV PCR-positive tumour of patient L31, the EBV PCR-positive tumour of patient L28, the HHV-6B and -7 PCR-positive tumour of patient L35, and the B19V PCR-positive tumour of patient L36. We applied RISH on 5-µm thick FFPE sections on SuperFrost glass slides with RNAscope 2.0 HD Red Chromogenic Reagent Kit (ACD) with probes targeting the regions *BILF-1* (catalogue Number 44781) of EBV, *U4* (870,801) of HHV-7; *U36* (521,401) of HHV-6B, *NS* (869,391) and *VP1* (872,961) of CuV and *NS1* (496,871) of B19V, according to the manufacturer’s protocol. The human housekeeping gene *PPIB* and the bacterial gene *DapB* (ACD) served as positive and negative technical controls, respectively.

### Immunohistochemistry (IHC) combined with RISH

To identify the host cells of CuV in the tumour of patient L31, we combined RNAscope in-situ hybridization (RISH) with fluorescent multiplex immunohistochemistry (mIHC) on 5-mm FFPE tissue sections, with multiple cellular markers on the same tissue section. The CuV probe (Probe-V-CuV-gp3-gp4-gp5 [NC_039050.1 (nt 2023-3562)], targets the sense DNA strand and mRNA of the *VP* gene region [15]. Probes targeting the human housekeeping gene *PPIB* and the bacterial gene *DapB* (ACD) served as positive and negative technical controls, respectively.

**Fig. 1:**
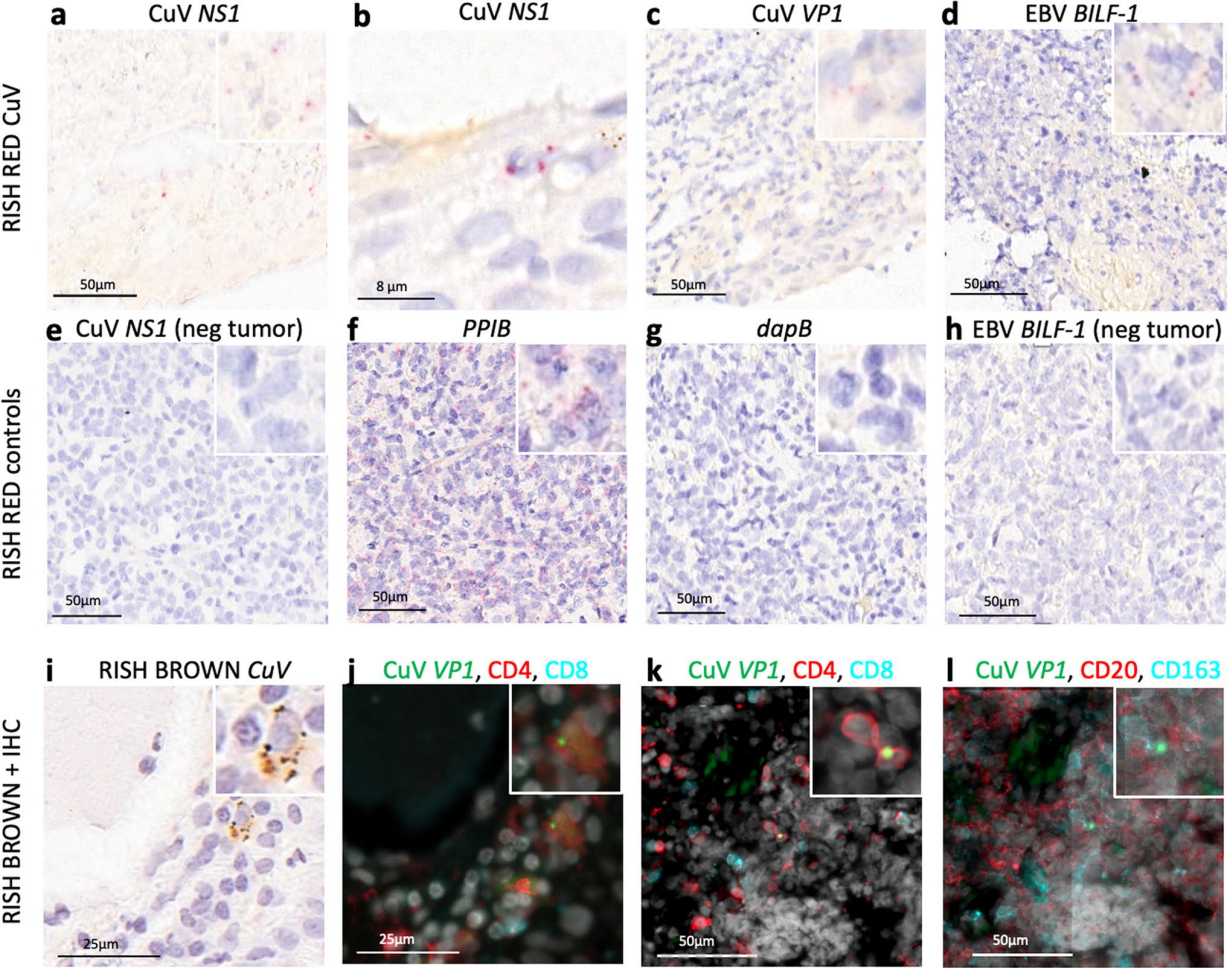
Virus RNAscope in situ hybridisation and cell-marker immunohistochemistry on FFPE tissues of two SL patients. RNAscope 2.5 Assay-RED (**a**–**h**) or Assay-BROWN (**i**) in combination with the following CD markers in IHC (**j**–**l**): CD4, CD8, CD20, and CD163, as below. Counterstained with hematoxylin. Red (**a**–**h**), brown (**i**), and green (**j**–**l**) punctuated dots represent positive RISH signals. **a** CuV *NS1* RISH for CuV PCR-positive SL section. **b** CuV *NS1* RISH, same section as a., another area magnified. **c** A consecutive section, same histological area as in a., stained with CuV *VP1*. d. EBV *BILF-1* RISH for qPCR EBV-positive SL section. e. CuV *NS1* RISH for CuV PCR-negative SL section. **f** Human *PPIB* mRNA probe (positive control). **g**. Bacterial *dapB* probe (negative control). **h** EBV *BILF-1* RISH for EBV PCR-negative SL section. **i** CuV *NS1* RISH Brown for CuV PCR-positive SL section. **j** A consecutive section, same histological area as in (**i**), stained for CD4 + (red) and CD8 (light blue) T cells after CuV *VP1* RISH (green punctuated dots). **k** Same section from different area as in (**j**.), stained for CD4 + (red) and CD 8 + (light blue) T cells after CuV *VP1* RISH (green punctuated dots). **l** Same section and histological area as in (**k**), with a consecutive staining for CD20 + (red) B cells and CD163 (light blue) macrophages. Light grey stain shows nuclear DAPI

RISH (RNAscope 2.5 HD reagent kit-BROWN; [PN 322310; ACD, Newark, USA]) was employed according to the manufacturer’s protocol with slight modification. After addition of the AMP6 reagent, Alexa Fluor™ 488 Tyramide Reagent (Thermo Fisher [cat: B40953]) was applied to the tissue sections in a 1:100 dilution for 5 min, to make the RISH signal fluorescent. This was then followed by mIHC, as previously described (Blom et al., 2017), with some modifications. Briefly, primary antibodies raised in different species were applied pairwise in sequential staining rounds and detected by AlexaFluor647 and AlexaFluor750 fluorochrome-conjugated secondary antibodies. The following primary biomarker antibodies (1:200): helper T cells, (Rabbit-anti-CD4; Abcam; ab133616), cytotoxic T cells (Mouse-anti-CD8; Dako; M7103), macrophages (Mouse-anti-CD163; Thermo; MS-340) and B cells (Rabbit-anti-CD163; Abcam ab188571). DAPI was applied as a nuclear stain. After each round of staining the tissues were scanned on a Zeiss Axioscan.Z1 slide scanner. Before the next round of staining, coverslips were soaked off in wash buffer and the tissues were incubated in a bleaching solution (TBS/24 mM NaOH/4.5% H_2_O_2_) for 1 h at room temperature. Then the slides were heated in 10 mM Tris–HCl/1 mM EDTA, pH 9, for 20 min at 99°C to inactivate the primary antibodies of the previous staining round. Representative CuV RISH-mIHC- and RISH only-treated SL biopsy tissues are shown in Fig. 1.

### Parvovirus IgG enzyme immunoassay (EIA)

Seven of the 24 patients included in this study, had plasma samples stored at the Helsinki Biobank. Plasma samples but no FFPE tumour blocks were available from three additional patients with SL. We tested all the 10 plasma samples by in-house IgG EIAs, using biotinylated VP2 virus-like particles (VLP) as antigen for BuV, CuV and TuV, as well as for B19V, as previously described [33, 34]. The initial results for protoparvovirus IgGs were confirmed with a competition assay [33]. Optical densities (ODs) were measured at 450 nm (Multiskan EX; Thermo Fischer Scientific).

## Results

### Patient characteristics

Clinical information was available for 17 patients and unavailable for seven patients (Table 1). Overall, 50% of the patients were female. All the tumours were known to be situated in the sinonasal area, but the exact location of the tumour was not available for eight patients. In addition to SL DLBCL-NOS (NOS—not otherwise specified), patient L18 had a systemic T-cell lymphoma. Pathological reports were available for all patients, but in some cases the diagnosis did not follow the latest lymphoma classification due to old age of samples. The histological SL subtypes represented DLBCL-NOS (n = 16, 67%), mantle-cell lymphoma (MCL, n = 2, 8%), and smaller entities (Table 1). Immunostainings for EBV and ALK (anaplastic lymphoma kinase positive) were reported for some samples: samples L19 and L21 were EBNA2 and LMP1 negative, L25 was ALK unknown, L28 was positive and L34 was negative for EBER by chromogenic in situ hybridisation. It is noteworthy that there were no lymphomas of NK-cell subtype within this cohort.

The cohort included many patients with compromised immunity before the manifestation of SL (Table 1). Reasons such as malnutrition or tobacco smoking, were not reported in the patient charts.

### Presence of virus DNA by qPCR

We detected virus DNA in 15/24 (63%) tumours with viral loads ranging between 4.4 and 2.1 × 10^6^ copies per one million cells (cpm) (Table 3). Nine tumours were positive for EBV DNA (38%; 3.6 × 10^1^–2.1 × 10^6^ cpm). The DLBCL-NOS subtype harboured EBV DNA in 6/16 (38%) of the tumours. Both MCLs were EBV-DNA positive as well as the single anaplastic tumour.

**Table 3:** Positive virus DNA findings in qPCR in order of prevalence (virus DNA copies/1 million cells), results of parvovirus EIA IgG, and lymphoma subtypes

Patient code	EBV	HHV-7	B19V	HHV-6B	CMV	CuV	MCPyV	EIA IgG (BuV, CuV, TuV, B19V) *	Lymphoma subtype
L11*	1.8E + 02	2.9E + 01	–	4.4E + 00	–	–	–	B19V	MCL
L14/15	4.1E + 02	–	1.9E + 03	2.2E + 02	–	–	2.1E + 02	N/A	DLBCL–NOS
L17	–	–	2.9E + 01	–	–	–	–	N/A	Centroblastic/diffuse large B cell
L18	2.5E + 03	–	–	–	–	–	–	N/A	DLBCL–NOS
L25	5.1E + 02	2.8E + 02	–	–	–	–	–	N/A	Anaplastic large cell
L27	1.4E + 03	4.1E + 01	–	–	–	–	–	N/A	DLBCL–NOS
L28*	2.1E + 06	–	–	–	8.7E + 02	–	–	N/A	DLBCL–NOS
L29	2.1E + 02	–	–	–	–	–	–	N/A	DLBCL–NOS
L30	–	1.1E + 02	9.6E + 01	–	–	–	–	N/A	DLBCL–NOS
L31*	–	–	–	–	–	3.1E + 05	–	CuV	DLBCL–NOS, BCL–rearrangement
L32*	9.2E + 02	4.2E + 01	–	–	1.6E + 02	–	–	B19V	MCL
L33	–	–	–	8.4E + 00	–	–	–	B19V	DLBCL–NOS
L34	3.4E + 01	–	–	–	–	–	–	negative	DLBCL–NOS
L35	–	4.8E + 02	–	3.5E + 02	–	–	–	BuV2	DLBCL–NOS
L36*	–	–	2.1E + 03	–	–	–	–	B19V	DLBCL–NOS

^*^Altogether 10 plasma samples were analysed, but only the ones that had a tumour tissue available were shown here. One additional sample was CuV IgG positive

*N/A* not analysed

Six tumours were positive for HHV-7 DNA (25%; 2.9 × 10^1^–4.8 × 10^2^ cpm), detected in almost all subtypes present in our study: DLBCL-NOS, MCL, and anaplastic lymphoma. We found B19V DNA and HHV-6B DNA in four tumours each (17%; 2.9 × 10^1^–2.1 × 10^3^ cpm; 4.4 × 10^1^–3.5 × 10^2^ cpm, respectively): B19V DNA in DLBCL-NOS, and HHV-6B in MCL, and DLBCL-NOS. We found CMV DNA in two tumours (8%; 1.6 × 10^2^–8.7 × 10^2^ cpm) representing MCL, and DLBCL-NOS, respectively; and detected CuV DNA (3.1 × 10^5^ cpm) and MCPyV DNA (2.1 × 10^2^ cpm) in one tumour each (4%): both DLBCL-NOS. The human single-copy *RNase-P* qPCR served for quantification of human cells with a variation of 2.3 × 10^3^–1 × 10^6^ (mean: 2.7 × 10^4^) cells/ul DNA extract. All positive findings were reproducible.

Two or more different viral DNAs were co-detected in 8/24 (33%) tumours with up to four different viruses per tumour (Table 3). EBV was co-detected with other viruses in 78% of EBV-positive tumour samples, most often with HHV-7. HHV-6B, -7, and CMV were never the only viral findings in tumours but were present in combinations with other viruses; with EBV in 22%, 44%, and 100%; with B19V in 17%, 25%, and 0%; and with MCPyV in 0%, 25%, and 0% of HHV-6B, HHV-7 or CMV-positive  SL samples, respectively.

Among the five patients that were immunocompromised before the onset of the lymphoma, we detected virus DNA in four. Two out of the three patients with HIV were EBV-DNA positive. A slight emphasis was noted in the overall virus DNA prevalence (63% vs 80%) and the co-detections (1.6 virus co-detections/tumour vs 1.8 co-detections/tumour), between our groups of healthy, and the previously immunocompromised individuals, respectively.

While the mean age of the  patients at the time of SL diagnosis was 66 years (range 34–86), the mean age at diagnosis for the virus DNA-positive and -negative groups was 62 years (SD ± 14, range 34–84) and 73 years (SD ± 12, range 46–86), respectively. However, the age difference was not statistically significant (p = 0.06).

### Presence of CuV DNA in blood plasma by NGS

As the relevance of CuV persistence is unknown and interesting, we analysed the plasma sample from the patient with CuV PCR-positive SL (SL31) with targeted NGS to investigate a possible viremia (Table 3). The plasma sample was NGS positive for CuV (seven unique reads). We could, however, not repeat the result of plasma CuV positivity with qPCR.

### Location of CuV, EBV, HHV-6B and HHV-7 in tissues

To investigate EBV, we chose a high copy-number DLBCL-NOS tumour (patient L28, AIDS positive, Tables 1, 3). The EBV probe *BILF-1* demonstrated scattered signals on a broad area of the tumour (Fig. 1d). In the CuV-positive tumour (patient L31), we detected repeatedly intense positive signals by both CuV probes *VP1* and *NS1*, matching with each other on the layout, with signals in single spots or small clusters spread out unevenly throughout the tumour (Fig. 1 a-c).

When combining RISH with IHC on the same slide, we could localize CuV in CD4+ T cells, however, with fewer recognizable positive CuV spots when compared to RISH alone (Fig. 1a–c and i–l). We did not detect CD8+ , B cells or macrophages overlapping or in direct contact with CuV signals.

To detect HHV-6B and -7, and B19V, we chose tumour samples with the highest, however still modest, virus loads (2.07 × 10^2^ cpm; patient L35 for both HHVs, 2.07 × 10^3^ cpm for B19V), without solid findings. PCR-negative tumour controls remained all negative.

### Parvovirus enzyme immunoassay

To search for signs of immune system activation, and to survey the seroprevalence of the ‘new’ protoparvoviruses, we performed protoparvovirus and B19V EIAs for all the 10 plasma samples available. Two out of the 10 plasma samples were positive for CuV-specific IgG (absorbance values 1.5 and 2.1). One of the CuV IgG-positive plasma samples belonged to patient L31, who was positive for CuV DNA both in FFPE tumour (qPCR) and in plasma (NGS). The other CuV IgG-positive plasma belonged to a SL patient with no FFPE-tumour sample available. One plasma sample was BuV2-IgG positive. The IgG prevalence of B19V was 60% (6/10).

## Discussion

We explored the presence of herpes-, parvo- and polyomaviruses in SL. Our results revealed multiple viruses present in SL, often simultaneously, potentially related to the anatomical location and sensitive methods. We explored the entity further by NGS, RISH, and parvovirus EIAs for in-depth analysis.

In epidemiological studies, the mean age of SL patients is 63–68 years [1, 2], corresponding to that of our patient cohort, with a tendency towards earlier onset of disease in the virus-positive patients. No previous comprehensive SL virus-screening studies exist, but the virus-related tendency of younger age at disease onset is evident in other virus-associated tumours, such as HPV-positive oropharyngeal carcinoma and carcinoma ex pleomorphic adenoma of the salivary glands [35, 36].

We detected viral DNA in 63% of the 24 SL tumours. EBV DNA was the most prevalent virus finding (38%), followed by HHV-7 and -6B. These viruses are ubiquitous, and the primary infection occurs usually in early childhood or adolescence. EBV resides primarily in the B lymphocytes of the lymphatic system, the prevalence on the mucous membranes of the nose and nasopharynx varies between 0 and 10% [23, 37]. The EBV prevalence of 38% (6/16) in the DLBCL-NOS subtype observed in this study is highly above the range of the previously reported DLBCL EBV prevalence of less than 5% in the Western countries and of 4–14% worldwide [38, 39]. The high rate of EBV in our study could relate to the anatomical impact of the sinonasal tract compared to a systemic disease and the high sensitivity of PCR. 

The incidence rate of EBV positive DLBCL in patients with HIV-infection reaches up to 20–60% [38, 39]. HIV infection, especially at the AIDS stage, may predispose to malignant transformation of B cells into DLBCL, with EBV and HCV as important etiological factors [39, 40]. Our study supports these results. We noticed an accentuated EBV involvement in our previously immunocompromised patients with HIV, HCV, or with additional primary malignancies, with an EBV-DNA positivity of 67% in their SL tumours. We were able to detect EBV throughout the tumour with RISH targeting *BILF-1*, a probe targeting a constitutively expressed G-protein-coupled receptor essential for EBV-mediated immunosuppression and oncogenesis [41], throughout the tumour with RISH (Fig. 1d).

In our cohort both sinonasal MCLs harboured EBV DNA. This unexpected finding contrasts with those of Carvalho et al. [42], where EBV ISH was negative in all 20 MCLs.. The discrepancy may be explained by the anatomical location, low virus loads (1.8 × 10^2^–9.2 × 10^2^ cpm) coupled with high sensitivity of our qPCR, and the biological diversity of MCL [42, 43]. The association between EBV and MCL remains unresolved and would benefit from larger mantle cell SL series with a high-sensitive approach.

As for the other members of the *Orthoherpesviridae*, HHV-7 DNA was detected in 25%, HHV-6B DNA in 17% and CMV DNA in 8% of the SL tumours. This is in line with the results of Hernandez-Losa et al. in 2005 with 15%, 27%, 4%, of HHV-7, -6B and CMV, respectively, in fresh-frozen tissue samples of any lymphoma subtypes [44]. Within healthy individuals, predominately HHV-7, and to a lesser extent HHV-6, were nearly always present in oral, and in approximately 50% of nasal swab samples [23]. Unfortunately, RISH targeting the *U38* gene of HHV-6B, the product of which is associated with active replication, and the *U4* gene of HHV-7 (unknown function), was not consistent probably due to low viral DNA loads or lack of active replication (Table 3).

Like other herpesviruses, HHV-6B and -7 establish life-long persistence and especially HHV-6B can reactivate in immunocompromised patients [21]. Proteins coded by HHV-6 and -7 have immunomodulatory functions by acting on HLA class I expression and chemokine receptors and thereby may weaken virus-specific immune responses by creating a microenvironment that favours viral persistence [21]. In addition, HHV-6 might facilitate and enhance the infections of HIV, HCMV, and EBV [21, 44, 45]. It is noteworthy, that EBV was almost exclusively detected in combination with other viruses, usually HHV-6B or -7 (Table 3). Two out of three patients with HIV were positive for HHV-6B or -7 as well. Therefore, the role of other viruses, such as HHV-6B and -7, could have an assisting role in disease development, but this would require larger studies for verification.

CuV is a rather recently discovered protoparvovirus [12], present in a relevant proportion of skin biopsies of cutaneous T-cell lymphoma (CTCL) 8.5–38% [13–15]. Therefore, we explored one DLBCL patient with a high CuV load (3.06 × 10^5^ cpm) in the FFPE tumour. We detected CuV DNA both in the SL tumour by qPCR (Tables 1, 3) and in the plasma by NGS, showing CuV DNAemia/viremia, previously reported only in one highly immunocompromised patient [46]. We did, however, not detect CuV DNA in the plasma by our qPCR, probably due to fragmentation or mismatches in the critical primer-or probe-binding areas. RISH was positive with both CuV probes NS1 and VP1 (Fig. 1a–c). We combined chromogenic RISH with fluorescent IHC, and CuV positivity was detected in CD4+ T cells, but not in B cells or CD8+ T cells. While no definitive conclusions can be made, our findings support the idea of CuV being linked to T cells in accordance with the finding of CuV detected in CTCL [13, 14]. Further, we detected CuV IgG in 2/10 (20%) of our SL plasma samples, which is interesting when compared to the low seroprevalence in the general population of below 6%, but similar to the higher seroprevalences of 9.5% and 33% in CTCL patients [14, 17, 33]. However, our sample size is too small to make firm conclusions about the prevalence.

There are some limitations to this study. Although we have been able to demonstrate the efficient use of FFPE tumour samples in virus, DNA screening [15, 29, 36], the harsh formalin fixation process harms the DNA and may interfere with the discovery of viruses. Small tumour sample sizes and low virus loads may further affect the results. To detect virus activity, RNA studies from fresh-frozen samples would be essential. In addition, the low volume and heterogeneity of our tumours are evident, and since both diagnostics and classification of lymphomas evolve continuously, the exact subtyping according to the latest classification was not possible in this study. In addition, clinical detailed data were lacking for older patients, which could affect the results and their interpretation, especially when discussing the characteristics of virus prevalence within certain lymphoma subtypes. Neither does our cohort represent a ‘typical’ setting of lymphomas, since there were no NK-T-cell lymphomas present.

## Conclusion

SLs constitute a rare entity of various lymphomas in a distinct anatomical site with proximity to a massive microbial load. The association of both DLBCL and MCL with EBV were highlighted in our cohort. The role of CMV, HHV-6 and -7, with recurring findings in SL, may provoke a disease-friendly microenvironment. The high viral loads in qPCR of a newcomer in virus research, CuV, is an intriguing finding of this study, with positive viral signals in RISH, CuV-DNA positivity in plasma, and the putative high CuV IgG seroprevalence among SL patients compared to the general population. Larger sample series focusing on aspects of virus activity would be an essential next step.

The mere presence of a virus is not proof of a causal association with disease development. However, persisting viruses might still be able to affect the cell [47]. The behaviour of the recently discovered viruses is unknown, and therefore, all insights are beneficial for future research.

## Data Availability

The datasets generated during and/or analyzed during the current study are not publicly available due to the sample size and rarity of these tumors. Data are available from the corresponding author on reasonable request.
